# Spatial Alignment for Unsupervised Domain Adaptive Single-Stage Object Detection

**DOI:** 10.3390/s22093253

**Published:** 2022-04-23

**Authors:** Hong Liang, Yanqi Tong, Qian Zhang

**Affiliations:** College of Computer Science and Technology, China University of Petroleum (East China), Qingdao 266555, China; liangh@upc.edu.cn (H.L.); zhangqian8266@163.com (Q.Z.)

**Keywords:** domain adaptation, object detection, transfer learning

## Abstract

Domain adaptation methods are proposed to improve the performance of object detection in new domains without additional annotation costs. Recently, domain adaptation methods based on adversarial learning to align source and target domain image distributions are effective. However, for object detection tasks, image-level alignment enforces the alignment of non-transferable background regions, which affects the performance of important target regions. Therefore, how to balance the alignment of background and target remains a challenge. In addition, the current research with good effect is based on two-stage detectors, and there are relatively few studies on single-stage detectors. To address these issues, in this paper, we propose a selective domain adaptation framework for the spatial alignment of a single-stage detector. The framework can identify the background and target and pay different attention to them. On the premise that the single-stage detector does not generate region suggestions, it can achieve domain feature alignment and reduce the influence of the background, enabling transfer between different domains. We validate the effectiveness of our method for weather discrepancy, camera angles, synthetic to real-world, and real images to artistic images. Extensive experiments on four representative adaptation tasks show that the method effectively improves the performance of single-stage object detectors in different domains while maintaining good scalability.

## 1. Introduction

Object detection is a fundamental and widely used topic in the field of computer vision. In the past few years, deep neural networks (DNNs) have achieved satisfactory results [1,2,3,4,5,6] in the presence of large amounts of labeled data. However, most of these studies are based on supervised learning methods, assuming that the training data and the test data have the same distribution, which obviously cannot always be satisfied in practical applications [7]. Because the real environment is uncontrollable, changes in environmental conditions, camera angles, and shooting distances may cause domain shifts, resulting in the trained model being unable to achieve expected results on other test sets. This hinders the deployment of the model in a real environment.

An obvious solution is to retrain with data collected in a new environment. Unfortunately, real-world environments usually do not have corresponding annotated images, and finely annotating each object instance in each image is usually expensive and time-consuming. Unsupervised domain adaptation (UDA) [8], assuming that there is no labeled data available in the target domain, is an attractive alternative to guide the model to transfer knowledge from the source domain to the target domain only if there is labeled data in the source domain.

In recent years, domain adaptation has achieved remarkable success in many fields, and mainstream methods adopt the idea of generative adversarial to align the feature distributions of source and target domains through adversarial learning [9,10,11]. In unsupervised object detection domain adaptation, DA Faster R-CNN [9] considers both image-level alignment and instance-level alignment. However, image-level alignment treats an image as a whole and does not distinguish between objects and backgrounds, whereas object detection tasks inherently focus on local regions that may contain objects of interest. Although ROI-based [12,13] instance-level alignment can match object proposals in both domains, the problem of forcing alignment of non-transferable background regions remains unsolved. Other methods improve DA Faster R-CNN [14,15,16,17], but these studies are all based on two-stage detectors, single-stage detectors directly perform localization and classification without generating regions of interest, thus these methods cannot be directly applied. In addition, previous methods only consider the invariant features of the learning domain or restrict the specific features of the source domain, ignoring the specific features of the target domain, but the ultimate goal is to achieve the best results on the target domain.

**Figure 1:**
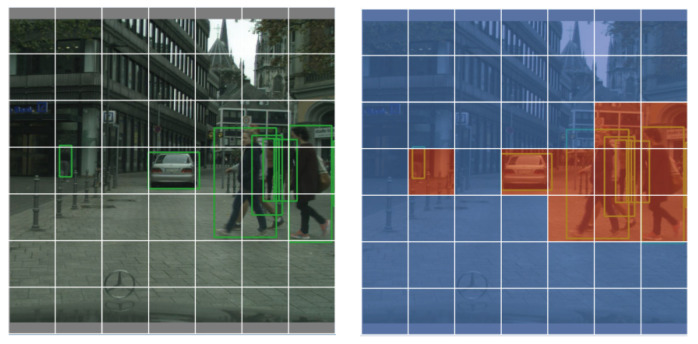
Schematic diagram of spatial domain alignment. Our spatial alignment framework enables more accurate alignment of background and important instances, which can help the backbone network to more accurately activate the main objects of interest in both domains and lead to better adaptive detection performance.

In this work, we address the problem of adaptively aligning background and instance features for single-stage detectors., and propose a new method suitable for single-stage object detection. The goal of this paper is to build an end-to-end single-stage domain-adaptive deep learning model based on YOLOv5, the key idea of which is to apply different attention to the background and target features when aligning them, setting different domain confidences for background and target, so that the network pays more attention to the target area and reduces the interference of the background, as shown in Figure 1. Since the target domain data does not have ground-truth boxes, we introduce a self-training [18,19] method to obtain pseudo-labels of the target domain data to guide domain alignment and assist training. In addition, the high-confidence output will lead to a large number of targets that cannot be accurately identified. To reduce the impact of false-negative samples in self-training, false-negative suppression loss (FNS) is used in the target domain to replace the original target loss.

The contributions of this work mainly include the following three aspects:

1. We propose a domain adaptation framework suitable for single-stage detectors, which combines image-level alignment and instance-level alignment. Balances the domain offset by applying different attention to the background and target through spatial domain alignment (SDA) and spatial consistency regularization (SCR);
2. We improve a new target loss in the target domain loss calculation, reducing the negative impact of false-negative samples by suppressing the loss of false-negative samples.
3. We validate the effectiveness of our method from four different perspectives (weather difference, camera angle, synthetic to real-world, and real images to artistic images). Experimental results show that this method can effectively improve the cross-domain detection performance of single-stage object detectors. Additionally, almost no increase in inference time and real-time detection can be achieved on GPU.

## 2. Related Work

Object detection—The task of object detection is to find out the categories and positions of all objects of interest in an image, which is one of the core problems in the field of computer vision. Object detection algorithms based on deep learning are mainly divided into two categories: two-stage detectors and single-stage detectors. The two-stage detector first performs region generation to obtain region proposals and then performs sample classification in the second stage through a convolutional neural network, such as R-CNN [2], Fast R-CNN [12], Faster R-CNN [13], R-FCN [4], etc. One-stage detectors extract features directly in the network to predict object classification and location. Common single-stage target detection algorithms include YOLO [20,21,22], SSD [23], and RetinaNet [24]. Compared with two-stage detectors that focus on accuracy, in recent studies, single-stage detectors focus on speed while maintaining acceptable accuracy, making the prediction effect comparable to two-stage object detection algorithms, which have become a popular paradigm. This paper selects YOLOv5 as the base detector, which enriches the research on domain adaptation of single-stage detectors.

Unsupervised domain adaptation–Unsupervised domain adaptation [25,26,27,28,29] is a frontier research direction within the field of computer vision and transfer learning, which attempts to transfer knowledge from one domain to another by mitigating distributional changes to improve deep neural network models across domains performance in tasks. Earlier studies were based on statistical difference minimization methods, which tried to measure and minimize the distances of features from different domains in a feature space, such as maximum mean discrepancy (MMD) [30], Wasserstein distance [31], Kullback–Leibler divergence (KL) [32], etc. In recent years, methods based on adversarial learning have gradually become the mainstream framework for UDA, and a lot of research has been done on it. The main idea of adversarial domain adaptation is to confuse the domain by adding a domain discriminator and gradient inversion layer during training, learn domain-invariant features that perform well on both source and target domains, and reduce the impact of domain offset. In addition, some methods have explored domain adaptation methods from other perspectives, such as using a style transfer network to reduce the differences between different domains and improve the effect of domain adaptation.

Domain adaptation in object detection—Domain adaptation has achieved satisfactory results in the fields of image classification and semantic segmentation. However, domain adaptation research in object detection is still in its early stages. Following the widespread success of adversarial learning-based methods in other domains, many adversarial-based domain adaptation frameworks have emerged in object detection. DA Faster R-CNN [9] is the first to introduce an adversarial learning-based method into object detection domain adaptation to achieve image-level alignment and instance-level alignment to reduce image-level and instance-level distribution differences. On this basis, recent studies have made a series of improvements to DA FasterR-CNN. Specifically, Xu et al. [33] proposed a classification regularization framework to achieve cross-domain matching of key image regions and important instances, and Saito et al. [8] proposed a strong local alignment and weak global alignment-based detector to help DA Faster R-CNN focus on aligning key regions and important objects across domains. Wang et al. [34] divided the weight direction and gradient into a domain-specific part and a domain-invariant part and removed the interference of the specific domain direction while learning the domain-invariant direction. Besides, Deng et al. [35] proposed an unbiased mean teacher (UMT) model, and achieved state-of-the-art results for various benchmarks of object detection.

## 3. Proposed Methods

In this section, we detail the technical details of the proposed method, adopting YOLOv5 as the baseline.

### 3.1. Framework Overview 

**Figure 2:**
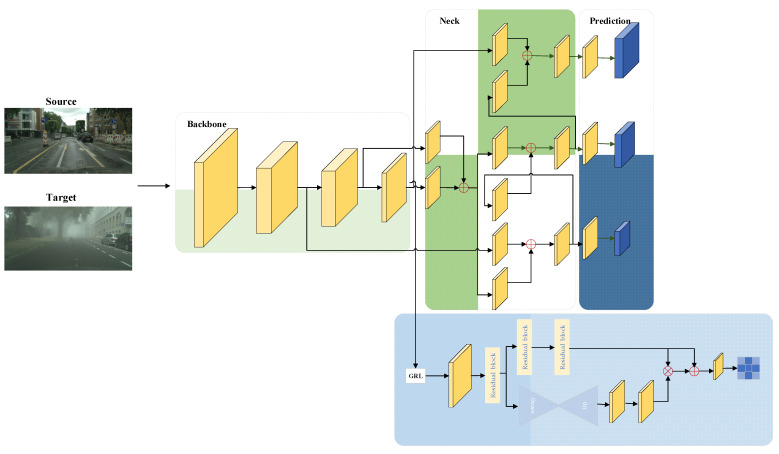
The overall structure of our framework. In this method, the spatial domain alignment module is added after the backbone network to predict the domain confidence of different locations and align the target and background effectively.

Our research involves scenes from two domains, a source domain with images and annotations (i.e., bounding boxes and target categories) and a target domain with only images. The final task is to use data from both domains to train a detector that performs well on the target domain. To this end, we propose a framework for selective domain adaptation based on spatial alignment, using YOLOv5 as the baseline. The overall architecture of the proposed method is shown in Figure 2. Aiming at the inconsistency of background and instance alignment requirements, we propose a spatial domain alignment module that balances the influence of background and target to better achieve domain feature alignment. In the training process, this module can guide the model to learn domain invariant features and reduce the gap between domains through backpropagation. Considering that each region of an image should come from the same domain, that is, image domain classification consistency, spatial alignment consistency regularization is added.

Our framework does not rely on RPN to extract regions of interest, thus it can be directly applied to single-stage detectors to better align background and targets across domains and improve adaptive detection performance. Additionally, our framework is flexible and generalizable and does not depend on specific algorithms for image or instance alignment. The training loss of the network is the sum of each part, which can be written as:

(1) $$L=L_{det}+\lambda \left(L_{d}+L_{cst}\right)$$

where λ is a trade-off parameter to balance YOLOv5 detection loss and newly added domain adaptive components. *L_det_* is the detection loss and *L_d_* and *L_cst_* are the domain identification loss and the domain consistency regularization loss, respectively. The network can be trained end-to-end. In addition, the antagonistic training of the domain adaptation component is realized by using a gradient reversal layer (GRL), which automatically reverses the gradient in the process of propagation. During inference, only the detection branch prediction results need to be used.

We detail the technical details of the proposed method below.

### 3.2. Spatial Domain Alignment Module

We utilize the improved domain classifier for feature alignment of the source and target domains. Generally speaking, the domain classifier judges the input as a whole from the source domain or the target domain and then conducts domain obfuscation through a gradient reversal layer (GRL) to achieve feature alignment. As mentioned earlier, it is difficult and unnecessary to achieve alignment of background and target of interest using the same settings. Aligning the object of interest in the feature space is more important than aligning the background. To overcome this problem, the spatial domain alignment module is designed to align the background and instances of an image. Specifically, we introduce a domain classifier after the YOLOv5 backbone network, through the gradient reversal layer, the gradient direction is automatically reversed in the backpropagation process, and the identity transformation is realized in the forward propagation process, and then a series of volumes are performed. The product and downsampling finally obtain the feature output of the m × m dimension. Each value represents the domain confidence for that location. In addition, a residual attention module is added to the domain classifier to better extract spatially effective information.

For each image, we wish to identify important regions of the object of interest and reassign the region representations. Single-stage detectors do not have region proposals from RPN, and a natural idea is to use the final predictions. We assume that x_s_, x_t_ are images from source domain S and target domain T, respectively, and y_s_, y_t_ are source domain label data and model-predicted pseudo-label data, respectively. According to (x_s_, y_s_), (x_t_, y_t_), the position of the target in the picture can be calculated. Then we divide the image into m × m grids, and assign attention to the corresponding positions according to the area where the target falls into the grid. We use the IOU as a measure, if the IOU is greater than 1/3, then the grid prediction is considered as foreground, otherwise, it is background. Different values for background and foreground are set as ground truth. Domain-consistent features are better learned by optimizing the domain classification loss to confuse the source and target domain features. We let *D_i,j,k_* denote the domain label of the *i*-th training image at the *j*-th row and the *k*-th column, 0 for the source domain target, 0.3 for the source domain background, 1 for the target domain target, and 0.7 for the target domain background. By denoting the output of the domain classifier as *p_i,j,k_* and using the cross-entropy loss, the spatial domain identification loss can be written as:

(2) $$L_{d}=\underset{i,j,k}{\sum }\;\left(D_{i,j,k}log\;p_{i,j,k}+\left(1-D_{i,j,k}\right)log(1-p_{i,j,k}\right))$$

### 3.3. Spatial Consistency Regularization

We assume that the source domain sample distribution is P_s_ (C, B, I) and the target domain sample distribution is P_t_ (C, B, I), where I is the image representation, B is the bounding box of an object, and C represents the class of the object. Because of the domain offset, P_s_ ≠ P_t_. We then use *P_i,j,k_* to represent the grid feature distribution of the *i*-th image row, the *j*-th row, and the *k*-th column of the image. D_i_ represents the feature distribution of the *i*-th image. Since it comes from the same image, there should be *P_i,j,k_* = *D_i_*. Therefore, we can alleviate the prediction bias of different regions of the same image by strengthening the spatial domain classification consistency. The spatial consistency regularization (SCR) can be written as:

(3) $$L_{cst}=||\frac{1}{N}\underset{i,j,k}{\sum }D_{i}-P_{i,j,k}|{|}_{2}$$

Among them, *N* represents the total number of image division areas, and ||.||_2_ represents the L2 regularization.

### 3.4. False-Negative Suppression Loss

In the target domain, to enable the model to learn the features of the target domain, guided learning with high model prediction confidence is used. In our attempts, directly applying the detection loss to the target domain would result in a negative gain, which is since wrong negative samples would lead to a reduced model recall, we minimize the impact of false negatives by modifying the loss in the target domain, and further propose to stabilize the training of the model with false-negative suppression (FNS) loss. According to the definition in YOLO, the original loss is defined as:

(4) $$L_{det}=L_{box}+L_{cls}+L_{obj}$$

where the object loss *L_obj_* is defined as:

(5) $$L_{\mathrm{obj}}=\lambda_{\mathrm{noobj}}\overset{S^{2}}{\underset{i=0}{\sum }}\overset{B}{\underset{j=0}{\sum }}1_{i,j}^{\mathrm{noobj}}{\left(ci-\hat{c}i\right)}^{2}+\lambda_{\mathrm{obj}}\overset{S^{2}}{\underset{i=0}{\sum }}\overset{B}{\underset{j=0}{\sum }}1_{i,j}^{\mathrm{obj}}{\left(ci-\hat{c}i\right)}^{2}$$

YOLOv5 judges whether there is a target in each prediction frame according to the label information and certain rules. If there is, the value of the corresponding position in the mask matrix is set to 1, otherwise, it is set to 0; obtaining the mask matrix of the image. The classification loss and bounding box loss are only calculated for the predicted box whose position in the mask matrix is 1. Additionally, all prediction boxes need to calculate the target loss, which will cause false-negative samples in the target domain to suppress the prediction that there is a target at that location. We can use the mask matrix to eliminate this effect and improve the target object loss as:

(6) $$L_{\mathrm{obj}}=\lambda_{\mathrm{obj}}\overset{S^{2}}{\underset{i=0}{\sum }}\overset{B}{\underset{j=0}{\sum }}1_{i,j}^{\mathrm{obj}}{\left(ci-\hat{c}i\right)}^{2}$$

## 4. Experiments

In this section, we introduce the details of typical datasets and implementation of domain adaptation, from weather discrepancy, camera angles, synthesis to the real-world discrepancy, and real images to artistic images to validate our approach; and compare our results with other methods. Furthermore, the effectiveness of the proposed method is verified by a simple ablation experiment.

### 4.1. Datasets and Evaluation 

Cityscapes [36]—The Cityscapes dataset is a large-scale urban streetscape dataset collected from different cities in normal weather. It contains 2975 training images and 500 validation images.

Foggy Cityscapes [37]—The Foggy Cityscapes dataset is made by adding synthetic fog based on the Cityscapes dataset. The synthetic haze transmittance is automatically generated, inheriting the semantic annotation of the original image. Each image in the Cityscapes will have three density levels of fog added, thus it contains 8925 training images and 1500 validation images.

KITTI [38]—KITTI is one of the most important datasets in the field of autonomous driving. Its images were collected from rural, urban, and highway areas. KITTI contains 7481 annotated images. We randomly select two-thirds of the images for training and other images for verification.

SIM10K [39]—SIM10K is a synthetic dataset generated by the Grand Theft Auto (GTAV) engine. It contains 10,000 images and 58,071 bounding box annotations, only cars are in this category. All images of SIM10K are utilized as the source domain.

PASCAL VOC [40]—The PASCAL VOC dataset is a dataset collected from the real world, which can be used for detection and segmentation. For detection tasks, it mainly includes 20 categories. We used the training set and verification set of PASCAL VOC 2007 and 2012 consisting of about 15 k images for training.

Clipart and Watercolor [41]—Clipart and Watercolor are artificial artistic images. Clipart contains 1000 images, a total of 20 categories. Watercolor has 2000 images in 6 categories.

### 4.2. Implementation Details

In all experiments, we use YOLOv5-l as the baseline, keeping the default training settings and hyperparameters of YOLOv5. The input image size is adjusted to 640 × 640 by scaling and padding, and stochastic gradient descent (SGD) is used for training. The initial learning rate is 0.01, the weight decay is 0.0005, and the SGD momentum is 0.937. During the experiment, the data of two domains are loaded for training, and the source domain and target domain are scrambled before training. Pseudo-labels are updated for each iteration during training. In the initial 100 iterations α = 0, the target domain only computes the domain classification loss, after 100 iterations, α gradually increases. For all experiments, we evaluate the mean precision with a threshold of 0.5 for comparison with other methods.

### 4.3. Experimental Results

In this section, we evaluate the object detection of our proposed spatially adaptive model in four different domain transfer scenarios: weather discrepancy (Cityscapes → Foggy Cityscapes), camera angle (Cityscapes → KITTI), synthetic to real-world discrepancy (SIM10K → Cityscapes), and real to artistic (PASCAL VOC → Clipart and Watercolor).

#### 4.3.1. Weather Discrepancy

Setting—In this section we use the Cityscapes dataset for clear weather and the Foggy Cityscapes dataset for foggy weather to study the adaptation of weather discrepancy; taking Cityscapes containing images and annotations as the source domain, and Foggy Cityscapes containing only images as the target domain. The source domain and target domain data are scrambled when reading to participate in the training process. It is finally evaluated on the validation set of Foggy Cityscapes.

Results—The comparison results are shown in Table 1. We used eight categories in our final evaluation. Since pre-training on different datasets has been shown to improve the generalization ability of the model, all methods are pre-trained on the COCO dataset for the convenience of comparison. Source only denotes the YOLOv5-l model trained on the source domain only. The results show that our method can improve the detection performance under different weather conditions, effectively solving the domain shift problem.

#### 4.3.2. Camera Angle

Setting—In this section, we use Cityscapes as the source domain and KITTI as the target domain to verify the performance of our method in scenarios with different camera configurations. During training, the training sets of the KITTI dataset and Cityscapes dataset are used. It is finally evaluated on the validation set of Cityscapes. The final evaluation used the only common class between the two domains—car.

Results—The results are shown in Table 2. It can be observed that our method achieves better performance in the end. Specifically, our method achieves an improvement of about 8.5%.

#### 4.3.3. Synthetic to Real

Setting—Since data acquisition can be difficult, the cost of sampling and labeling can be significantly reduced by generating synthetic data, thus this adaptation makes sense. During training, we use SIM10K as the source domain and Cityscapes as the target domain. It is finally evaluated on the validation set of Cityscapes. The evaluation of the results uses the only common class between the two domains—car.

Results—The final results are shown in Table 3. Experimental results demonstrate that our method can effectively utilize synthetic data and achieve domain adaptation from the synthetic to the real-world, reducing domain transfer.

#### 4.3.4. Real to Artistic

Setting—In this section, we will verify the effectiveness of adapting real images to artistic images. The source domain images are from Pascal VOC 2007 and 2012, and Clipart and Watercolor are used as the target domain, respectively. Finally, it is evaluated on the Clipart and Watercolor test set.

Results—The performance comparison of PASCAL VOC → Clipark and PASCAL VOC → Watercolor is shown in Table 4 and Table 5. Compared with the baseline, the proposed spatial selection domain alignment method improves the map on the Clipart and Watercolor target domain by 12.8% and 8.9%, respectively. The improved performance shows that our method is effective in realizing domain adaptation.

### 4.4. Ablation Experiment

In this section, to verify the effectiveness of different components in our method, thorough ablation experiments are performed. We design several variants of the model to verify the contributions of different components. The results are shown in Table 6. All experiments are based on the adaptation of Cityscapes to Foggy Cityscapes.

Table 6 shows that the various components of our proposed method are effective and complementary. Specifically, the performance of Ours-Type1, Ours-Type2, and Ours-Type7 shows that finer partitioning contributes to domain adaptation. Compared with Source Only, Ours-Type3 achieved a performance gain of +3.4% using the spatial domain alignment module. In addition, Ours-Type4 achieved an additional performance gain of +1.4% using only spatial consistency regularization (SCR), while Ours-Type5 achieved a gain of +5.5% using only FNS. Ours-Type7 combined with all components, reaches an average accuracy of 37.4%.

### 4.5. Visualization

**Figure 3:**
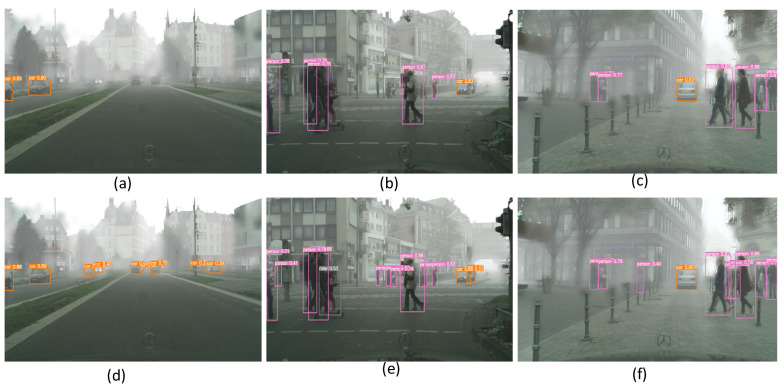
Object detection display. In the presence of large domain offsets, our spatially aligned selective domain adaptation framework enables YOLOv5 to produce more accurate detection results than training only on the source domain. (**a**–**c**): The detection results of the Source Only model. (**d**–**f**): The detection results of the proposed method.

We show an example of detection results in Figure 3. The first row of images is the result of training with only source domain images, illustrating the degraded performance of the detector when the distributions of the source and target domains do not match due to dense fog. The second row of images is domain-adapted using our method. We can see that our model locates the object correctly in these cases.

## 5. Conclusions

In this work, we investigate the alignment of background and object instances for unsupervised domain adaptation on a single-stage detector. Therefore, we propose a new UDA framework suitable for single-stage detectors with added attention to background and object instance settings for better feature alignment. Our key contribution is to address the problem of adaptively aligning background and instance features for single-stage detectors. We demonstrate the effectiveness of our approach from different perspectives, such as weather discrepancy, camera angles, synthetic data to the real-world images, and real images to artistic images. We conduct extensive experiments and ablation studies, which demonstrate that our method achieves competitive performance.

## Figures and Tables

**Table 1:** Weather adaptation: results on adaptation from Cityscapes to Foggy Cityscapes.

Cityscapes → Foggy Cityscapes									
Methods	Car	Person	Bicycle	Rider	Motorcycle	Bus	Truck	Train	mAP
Source Only	38.8	24.7	30.1	28.6	21.2	30.1	18.1	19.3	26.4
Two-stage methods									
DA-Faster-RCNN [9]	40.5	25.0	27.1	31.0	20.1	23.8	22.1	20.2	27.6
SWDA [10]	43.5	29.9	35.3	42.3	30.0	36.2	24.5	32.6	34.3
HTCN [14]	47.9	33.2	37.1	47.5	32.3	47.4	31.6	40.9	39.8
SCDA [15]	48.5	33.5	33.6	38.0	28.0	39.0	26.5	23.3	33.8
Faster-RCNN + ART + PSA [42]	52.1	34.0	37.4	46.9	34.7	43.2	30.8	29.9	38.6
MCAR [35]	43.9	32.0	36.6	42.1	37.4	44.1	31.3	43.4	38.8
MEAA [43]	52.4	34.2	36.2	48.9	33.2	42.7	30.3	46.0	40.5
UMT [44]	48.6	33.0	37.3	46.7	30.4	56.5	34.1	46.8	41.7
One-stage methods									
OSFA [45]	43.8	23.6	33.2	32.6	23.1	35.4	22.9	14.7	28.7
ST + C + RPL [46]	41.5	24.2	30.3	29.2	26.9	35.4	23.1	26.7	29.7
Ours	50.2	36.2	35.2	41.8	30.4	45.6	29.9	29.5	37.4

**Table 2:** Camera angle adaptation: results on adaptation from Cityscapes to KITTI.

Cityscapes → KITTI	
Methods	Car mAP
Source Only	37.7
DA-Faster-RCNN [9]	41.9
SCDA [15]	43.0
HTCN [14]	42.5
Faster-RCNN + ART + PSA [42]	41.0
Ours	46.2

**Table 3:** Synthetic-to-reality adaptation: results on adaptation from SIM10K to Cityscapes.

SIM10K → Cityscapes	
Methods	Car mAP
Source Only	36.8
DA-Faster-RCNN [9]	38.9
SCDA [15]	42.5
HTCN [14]	42.5
Faster-RCNN + ART + PSA [42]	43.8
MEAA [43]	42.0
Ours	42.6

**Table 4:** Real to artistic adaptation: results on adaptation from PASCAL VOC to Clipart.

PASCAL VOC → Clipart																					
Methods	Areo	Bike	Bird	Boat	Butt	Bus	Car	Cat	Chair	Cow	Tab	Dog	Horse	Mbike	Prsn	Plnt	Sheep	Sofa	Train	TV	mAP
Source Only	15.1	46.2	19.8	26.3	43.9	48.8	33.2	8.1	53.2	30.2	29.6	8.6	23.6	48.9	43.4	48.5	14.2	29.8	33.2	45.2	32.5
Two-stage methods																					
DA-Faster [9]	15.0	34.6	12.4	11.9	19.8	21.1	23.2	3.1	22.1	26.3	10.6	10.0	19.6	39.4	34.6	29.3	1.0	17.1	19.7	24.8	19.8
SWDA [10]	26.2	48.5	32.6	33.7	38.5	54.3	37.1	18.6	34.8	58.3	17.0	12.5	33.8	65.55	61.6	52.0	9.3	24.9	54.1	49.1	38.1
HTCN [14]	33.6	58.9	34.0	23.4	45.6	57.0	39.8	12.0	39.7	51.3	21.1	20.1	39.1	72.8	63.0	43.1	19.3	30.1	50.2	51.8	40.3
MEAA [43]	31.3	53.5	38.0	17.8	38.5	69.9	38.2	23.8	38.3	58.1	14.6	18.1	33.8	88.1	60.3	42.1	7.8	30.8	61.1	58.7	41.1
UMT [44]	39.6	59.1	32.4	35.0	45.1	61.9	48.4	7.5	46.0	67.6	21.4	29.5	48.2	75.9	70.5	56.7	25.9	28.9	39.4	43.6	44.1
One-stage methods																					
SSD + BSR + WST [17]	28.0	64.5	23.9	19.0	21.9	64.3	43.5	16.4	42.2	25.9	30.5	7.9	25.5	67.6	54.5	36.4	10.3	31.2	57.4	43.5	35.7
ST + C + RPL [46]	36.9	55.1	26.4	42.7	23.6	64.4	52.1	10.1	50.9	57.2	48.2	16.2	45.9	83.7	69.5	41.5	21.6	46.1	48.3	55.7	44.8
Ours	32.6	58.2	30.7	40.2	54.4	65.3	47.6	12.7	64.5	42.2	39.2	13.6	35.4	70.7	72.4	57.6	21.4	39.9	48.3	59.1	45.3

**Table 5:** Real to artistic adaptation: results on adaptation from PASCAL VOC to Watercolor.

PASCAL VOC → Watercolor							
Methods	Bike	Bird	Car	Cat	Dog	Person	mAP
Source Only	82.3	49.2	44.6	34.7	20.3	60.7	48.6
Two-stage methods							
DA-Faster [9]	75.2	40.6	48.0	31.5	20.6	60.0	46.0
SWDA [10]	82.3	55.9	46.5	32.7	35.5	66.7	53.3
HTCN [14]	87.9	52.1	51.8	41.6	33.8	68.8	56.0
MCAR [35]	87.9	52.1	51.8	41.6	33.8	68.8	56.0
MEAA [43]	81.0	53.2	54.0	40.1	39.2	65.3	55.5
UMT [44]	88.2	55.3	51.7	39.8	43.6	69.9	58.1
One-stage methods							
SSD + BSR + WST [17]	75.6	45.8	49.3	34.1	30.3	64.1	49.9
ST + C + RPL [46]	79.9	56.5	48.6	42.1	42.9	73.7	57.3
Ours	88.0	53.3	54.2	38.2	33.9	77.6	57.5

**Table 6:** Ablation study on adaptation from Cityscapes to Foggy-Cityscapes.

Methods	SCR	FNS	mAP
Source Only			26.4
Ours-Type1 (K = 1)	√	√	34.4
Ours-Type2 (K = 5)	√	√	36.7
Ours-Type3 (K = 7)			29.8
Ours-Type4 (K = 7)	√		31.2
Ours-Type5 (K = 7)		√	35.3
Ours-Type6 (K = 7)	√	√	37.4

## Data Availability

The data present in this study are openly available at https://ieeexplore.ieee.org/document/6248074/, accessed on 26 July 2012.
